# Graph-Based Audio Classification Using Pre-Trained Models and Graph Neural Networks

**DOI:** 10.3390/s24072106

**Published:** 2024-03-26

**Authors:** Andrés Eduardo Castro-Ospina, Miguel Angel Solarte-Sanchez, Laura Stella Vega-Escobar, Claudia Isaza, Juan David Martínez-Vargas

**Affiliations:** 1 Grupo de Investigación Máquinas Inteligentes y Reconocimiento de Patrones, Instituto Tecnológico Metropolitano, Medellín 050013, Colombia; miguelsolarte244621@correo.itm.edu.co (M.A.S.-S.); lauravega@itm.edu.co (L.S.V.-E.); 2 SISTEMIC, Electronic Engineering Department, Universidad de Antioquia-UdeA, Medellín 050010, Colombia; victoria.isaza@udea.edu.co; 3 GIDITIC, Universidad EAFIT, Medellín 050022, Colombia; jdmartinev@eafit.edu.co

**Keywords:** ecoacoustics, environmental sound classification, graph neural networks, graph representation learning, node classification, pre-trained models

## Abstract

Sound classification plays a crucial role in enhancing the interpretation, analysis, and use of acoustic data, leading to a wide range of practical applications, of which environmental sound analysis is one of the most important. In this paper, we explore the representation of audio data as graphs in the context of sound classification. We propose a methodology that leverages pre-trained audio models to extract deep features from audio files, which are then employed as node information to build graphs. Subsequently, we train various graph neural networks (GNNs), specifically graph convolutional networks (GCNs), GraphSAGE, and graph attention networks (GATs), to solve multi-class audio classification problems. Our findings underscore the effectiveness of employing graphs to represent audio data. Moreover, they highlight the competitive performance of GNNs in sound classification endeavors, with the GAT model emerging as the top performer, achieving a mean accuracy of 83% in classifying environmental sounds and 91% in identifying the land cover of a site based on its audio recording. In conclusion, this study provides novel insights into the potential of graph representation learning techniques for analyzing audio data.

## 1. Introduction

Graphs are powerful mathematical structures that have been extensively employed to model and analyze complex relationships and interactions across various domains [1]. In passive acoustic monitoring applications, which help to create conservation plans, ecoacoustics has recently gained great importance as a cost-effective tool to analyze species conservation and ecosystem alteration. In this field, it is necessary to analyze a large amount of acoustic data to assess variations in the ecosystem. Moreover, in recent years, the field of graph representation learning has grown due to the increased interest in using these graph structures for learning and inference tasks [2]. To learn from graphs, it is crucial to develop algorithms and models that can efficiently capture and make use of the detailed structural information present in graph data. These approaches have found applications in diverse fields, including bioinformatics, computer vision, recommendation systems, and social network analysis [3,4,5,6].

Graph neural networks (GNNs) have emerged as a prominent class of models for learning on graphs, offering distinct advantages over traditional artificial intelligence techniques [7]. Unlike traditional methods that operate on independent data points, GNNs use the inherent connectivity and dependencies within the graph structure to learn and propagate information across nodes. By recursively aggregating and transforming node features based on their local neighborhood, GNNs can capture both local and global patterns, enabling them to model complex relationships in graph data effectively. Notably, significant advancements in tasks such as node classification, link prediction, and graph generation have been made by leveraging their ability to capture structural dependencies [8].

Automatic audio classification tasks have attracted attention in recent years, specifically the classification of environmental sounds [9], enabling applications ranging from speech recognition [10,11] to soundscape ecology [12,13]. Traditional classification techniques such as *k*-nearest neighbors, support vector machines, and neural network classifiers have been used [14,15,16,17]. However, its performance mostly relies on hand-crafted features from representations as temporal, spectral, or spectro-temporal domains. Moreover, deep learning techniques using 1D (raw waveform) [18,19,20,21] or 2D (spectrograms) [22,23,24,25] convolutional neural networks (CNN) have shown significant improvements over hand-crafted methods. Nevertheless, these networks do not consider the relationships that may exist between different environmental sounds. Recurrent Neural Networks were initially proposed to capture feature dependencies from audio data [26,27,28]. More recently, Transformer models have emerged to model longer feature dependencies and leverage parallel processing [29,30,31,32]. Transformer models can handle variable input lengths and utilize attention mechanisms, making them aware of the global context and allowing their application on audio classification tasks.

Although graphs have been widely employed to represent and analyze visual and textual data, their potential to represent audio data has received relatively less attention [33,34,35]. Nonetheless, audio data, ranging from speech signals to music recordings, inherently exhibit temporal dependencies and complex patterns that can be effectively captured and modeled using graph-based representations. Working with graphs presents several challenges in their construction and subsequent processing. Determining how to generate feature information for each node and establishing connections between nodes in the network remain open problems. In this study, we propose utilizing pre-trained audio models to extract informative features from audio files, enabling the building of graphs that capture the inherent relationships and temporal dependencies present in the audio data.

Specifically, this study aims to address the problem of audio classification as a node classification task over graphs. To achieve this, we propose the following approach: (i) characterizing each audio with pre-trained networks to leverage transfer learning from models trained on large amounts of similar data, (ii) constructing graphs with each set of generated features, and (iii) utilizing the constructed graphs to classify nodes into predefined categories, taking advantage of their relationship. To accomplish this, we will use two datasets, a public one and one acquired in a passive acoustic monitoring study. We will evaluate the performance of three state-of-the-art GNNs: convolutional graph networks (GCN), graph attention networks (GAT), and GraphSAGE. These models leverage the rich structural information encoded in audio graphs in a transductive manner to learn discriminative representations capable of efficiently distinguishing between different audio classes. By comparing the performance of these models, we attempt to evaluate which of the graph models performs better on audio classification tasks.

In conclusion, this study contributes to the emerging field of graph representation learning by exploring the application of GNNs for audio classification. In particular, we demonstrate the effectiveness of pre-trained audio models to generate node information for graph representations and compare the performance of three GNN architectures. The results not only advance the state-of-the-art in audio classification but also emphasize the potential of graph-based approaches for modeling and analyzing complex audio data.

## 2. Graph Neural Networks

A graph is a widely used data structure, denoted as $G=\left(V,E\right)$, consisting of nodes $\left(V=\left\{v_{1},v_{2},\ldots ,v_{\left|V\right|}\right\}\right)$ and edges $\left(E=\{e_{ij}\}\right)$ representing a link between node $i\;\left(v_{i}\right)$ and node $j\;\left(v_{j}\right)$. A useful way to represent a graph is through an adjacency matrix $\left(A\in R^{\left|V\right|\times \left|V\right|}\right)$, where the presence of an edge is encoded as an entry with $A_{ij}=1$ if there is an edge between $\left(v_{i}\right)$ and $\left(v_{j}\right)$ and $A_{ij}=0$ otherwise. Additionally, each node *i* has associated feature information or embeddings denoted as $h_{i}^{\left(0\right)}$.

GNNs are machine learning methods that receive data in the form of graphs and use neural message passing to generate embeddings for graphs and subgraphs. In [2], the author provides an overview of neural message passing, which can be expressed as follows:

(1) $$h_{u}^{\left(k+1\right)}=update^{\left(k\right)}\left(h_{u}^{\left(k\right)},agg^{\left(k\right)}\left(\{h_{v},\forall \;v\in N\left(u\right)\}\right)\right).$$

In this equation, $h_{u}^{\left(k\right)}$ is the current embedding of node *u* where the embeddings ($h_{v}$) of neighboring nodes will be sent; $N\left(u\right)$, the neighborhood of node *u*; and $update^{\left(k\right)}$ and $agg^{\left(k\right)}$, permutation-invariant functions.

There exist various GNN models that differ in their approach to the *aggregation* or *update* function expressed in Equation (1) and in their ability to perform prediction tasks at node, edge, or network level [36]. The theory of the three GNN models used in this study is presented below.

### 2.1. Graph Convolutional Networks (GCNs)

The goal of GCNs is to generalize the convolution operation to graph data by aggregating both self-features and neighbors’ features [37]. Following the update rule given by Equation (2), GCNs enforce self-connections by making $\tilde{A}=A+I$ and stack multiple convolutional layers followed by nonlinear activation functions.

(2) $$H^{\left(k+1\right)}=\sigma \left({\tilde{D}}^{-\frac{1}{2}}\tilde{A}{\tilde{D}}^{-\frac{1}{2}}H^{\left(k\right)}W^{\left(k\right)}\right)$$

In this equation, *H* is the feature matrix containing the embeddings of the nodes as rows, and $\tilde{D}$ denotes the degree matrix of the graph, which is computed as ${\tilde{D}}_{ij}=\sum_{j}{\tilde{A}}_{ij}$. Moreover, σ(·) is an activation function, and *W* is a trainable weight matrix.

### 2.2. Graph SAmple and aggreGatE (GraphSAGE)

GraphSAGE, a framework built on top of the original GCN model [38], updates each node’s embedding information by sampling the number of neighbors at different hop values and aggregating their respective embedding information. This iterative process allows nodes to increasingly gain information from different parts of the graph.

The main difference between the GCN model and GraphSAGE lies in the aggregation function. Where GCNs use an average aggregator, GraphSAGE employs a generalized aggregation function. Also, in GraphSAGE, self-features are not aggregated at each layer. Instead, the aggregated neighborhood features are concatenated with self-features, as shown in Equation (3).

(3) $$h_{u}^{\left(k+1\right)}=\sigma \left(\left[W^{\left(k\right)}agg\left(\{h_{v}^{\left(k\right)},\forall \;v\in N\left(u\right)\}\right),B^{\left(k\right)}h_{u}^{\left(k\right)}\right]\right)$$

In this equation, *B* is a trainable weight matrix, and agg denotes a generalized aggregation function, such as mean, pooling, or LSTM.

### 2.3. Graph Attention Networks (GATs)

In GCNs (Equation (2)), graph node features are averaged at each layer, with weights determined by coefficients obtained from the degree matrix ($\tilde{D}$). This implies that the outcomes of GCNs are highly dependent on the graph structure. GATs [39], for their part, seek to reduce this dependency by implicitly calculating these coefficients, taking into account the importance assigned to each node’s features using the attention mechanism [40]. The purpose of this is to increase the model’s representational capacity.

The expression for GATs is presented in Equation (4).

(4) $$h_{u}^{\left(k+1\right)}=\sigma \left(\sum_{v\in N\left(u\right)}\alpha_{uv}W^{\left(k\right)}h_{v}^{\left(k\right)}\right)$$

In this equation, $\alpha_{uv}$ represents the attention coefficients of the neighbors of node *u*, $v\in N\left(u\right)$, regarding the aggregation feature aggregation at this node. These coefficients are computed as

(5) $$\alpha_{uv}=\frac{exp\left(a^{⊤}LeakyReLU\left(W\left[h_{u},h_{v}\right]\right)\right)}{\sum_{j\in N\left(u\right)}exp\left(a^{⊤}LeakyReLU\left(W\left[h_{u},h_{j}\right]\right)\right)},$$

with *a* denoting a trainable attention vector [41].

## 3. Materials

### 3.1. UrbanSound8K

UrbanSound8K is an audio dataset [42] that contains 8732 labeled audio files in WAV format and lasts four seconds or less. Each audio file belongs to one of the following ten classes: *air conditioner*, *car horn*, *children playing*, *dog bark*, *drilling*, *engine idling*, *gun shot*, *jackhammer*, *siren*, and *street music*.

**Figure 1:**
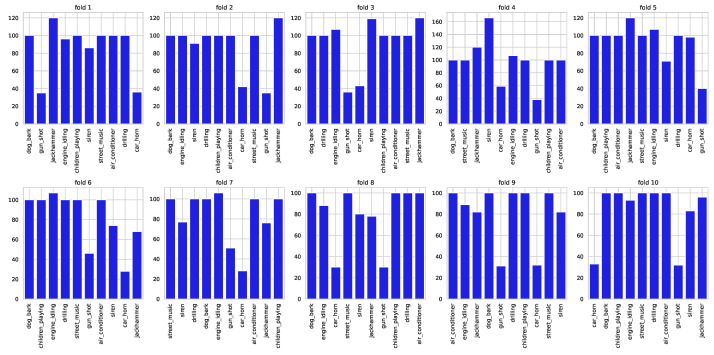
Distribution of the ten classes across the predefined folds.

The audio files are originally pre-distributed across ten folds, as depicted in Figure 1. To avoid errors that could invalidate the results and enable fair comparisons with existing literature, it is advised to perform cross-validation using the ten predefined folds.

### 3.2. Rey Zamuro Reserve

This dataset arises from a passive acoustic monitoring study conducted at Rey Zamuro and Matarredonda Private Reserves (3°31′02.5″ N, 73°23′43.8″ W), located in the municipality of San Martín in the Department of Meta, Colombia. The reserve covers an expanse of 6000 hectares, predominantly characterized by natural savanna constituting around 60%, interspersed with introduced pasture areas. The remaining 40% is covered by forests. This region falls within the tropical humid biome of the Meta foothills, showcasing an average temperature of 25.6 °C.

Data were acoustically recorded in September of 2022. A 13 × 8 grid was installed with 94 AudioMoth automatic acoustic devices placed 400 m from each other; of these recorders, one was not used due to deteriorated audio. The recording was made every fourteen minutes for seven consecutive days. The recordings were captured in mono format at a sampling rate of 192,000 Hz. The study encompassed various habitats, such as forest interiors, edges, and adjacent areas, each with distinct characteristics, including undergrowth. The recording heights were standardized at 1.5 m above the ground.

Depending on the kind of land cover, each acoustic recording of Rey Zamuro soundscapes was classified as forest, savanna, or pasture. These labels were given based on the placement of each automated recording unit. A total of 71,497 recordings were obtained, of which 14,546 correspond to forest class, 14,994 to savanna class, and 41,957 to pasture class. In all, 80% of the dataset is used as the training set, and the remaining 20% as the test set.

### 3.3. Pre-Trained Models for Audio Feature Extraction

We use pre-trained deep learning audio models to extract deep features from each audio file, which will be used as node information in the constructed graphs, i.e., as the values $h_{i}^{\left(0\right)}$. Specifically, we employed the following three models: VGGish, YAMNet, and PANNs.

#### 3.3.1. VGGish

VGGish is a pre-trained neural network architecture particularly designed to generate compact and informative representations, or deep embeddings, for audio signals [43]. It is inspired by the Visual Geometry Group (VGG) network architecture originally developed for image classification [44]. The deep embeddings generated by VGGish effectively capture relevant acoustic features and serve as a foundation for various audio processing tasks, such as audio classification, content-based retrieval, and acoustic scene understanding [45,46]. VGGish was trained on AudioSet [47], a publicly available and widely used large-scale audio dataset comprising millions of annotated audio clips and 527 classes, including animal sounds, musical instruments, human activities, environmental sounds, and more.

The architecture of VGGish consists of several layers, including convolutional, max-pooling, and fully connected layers. In this model, the processed audio is segmented into 0.96-second clips, and a log-Mel spectrogram is calculated for each clip, serving as the input to the neural network. Then, the convolutional layers apply a set of learnable filters to the input audio spectrogram, aiming to detect local patterns and extract low-level features. Following each convolutional layer, max-pooling layers are employed to reduce the spatial dimensions of the obtained feature maps while retaining the most important information. This process helps capture and preserve relevant patterns at different scales and further abstract the representations. Lastly, the final layers of VGGish, i.e., the fully connected layers, take the flattened output of the preceding convolutional and max-pooling layers and map it to a 128-dimensional representation. This mapping aims to capture global and high-level dependencies, resulting in deep embeddings that encode meaningful information about the audio signal and can serve as input for subsequent shallow or deep learning methods.

#### 3.3.2. PANNs

Large-scale Pretrained Audio Neural Networks (PANNs) are pre-trained models specifically developed for audio pattern recognition [48]. Their architecture is built upon CNNs, which are well-suited for analyzing audio mel-spectrograms. PANNs have multiple layers, including convolutional, pooling, and fully connected layers. These layers work together to learn hierarchical representations of audio patterns at various levels of abstraction.

The training process of PANNs involves pre-training the model on the large-scale AudioSet dataset. By being trained on this dataset, PANNs learn to capture a wide range of audio patterns, making them strong audio feature extractors. These audio patterns are then mapped to a 2048-dimensional output space.

#### 3.3.3. YAMNet

Yet another Audio Mobilenet Network (YAMNet) is a pre-trained neural network architecture that utilizes the power of deep CNNs and transfer learning to perform accurate and efficient audio analysis [49].

YAMNet is a mobilenet-based architecture consisting of a stack of convolutional layers, followed by global average pooling and a final fully connected layer with softmax activation. The convolutional layers extract local features by convolving small filters over the input audio spectrogram, thereby capturing different levels of temporal and spectral patterns. Then, the global average pooling operation condenses the extracted features into a fixed-length representation. Finally, the fully connected layer produces the classification probabilities for each sound class.

YAMNet’s primary objective is to accurately classify audio signals into a wide range of sound categories. However, the embeddings obtained after the global average pooling operation can also be useful.

To process audio, YAMNet divides the audio into segments of 0.96 s with a hop length of 0.48 s. For each segment, a feature output comprising 1024 dimensions is generated.

### 3.4. Graph Construction

A popular way to determine the edges of a graph is to define whether two points are neighbors through the *k*-nearest neighbors (*k*-NN) algorithm. According to this method, the neighbors of node $v_{i}$ are those *k*-nearest neighbors in the feature space [50]. Thus, the *k*-NN algorithm assigns edges between $v_{i}$ and its neighbors.

## 4. Experimental Framework

**Figure 2:**
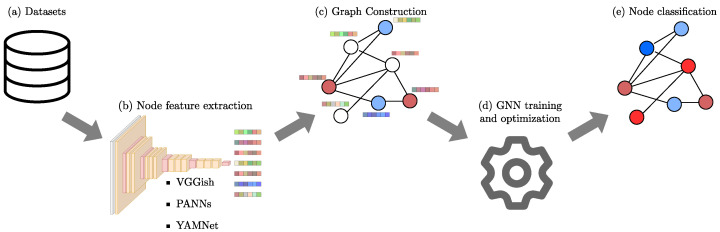
The workflow diagram proposed in this study illustrates that for each audio of a dataset (**a**) deep features are extracted with pre-trained audio models (**b**), then graphs are constructed by including those features as node information and setting edges with *k*-NN (**c**). For test data, the nodes present information but no labels (in the diagram the nodes unfilled are the test nodes). Subsequently, some GNN models are trained and optimized (**d**). Finally, trained models allow discriminating test nodes between classes (red or blue in the diagram) through transductive learning (**e**).

The proposed methodology of this study to assess the effectiveness of using graphs to represent audio data by leveraging pre-trained audio models to generate node information is depicted in Figure 2, and involves the following stages: (i) VGGish, YAMNet, and PANNs pre-trained audio models are used to extract features from both datasets, (ii) those deep features are used independently to construct graphs where each node represents an audio file, and edges are determined based on the *k*-NN algorithm, and (iii) the constructed graphs are used to train and optimize certain hyperparameters on GCN, GraphSAGE, and GAT models to perform node classification.

**Figure 3:**
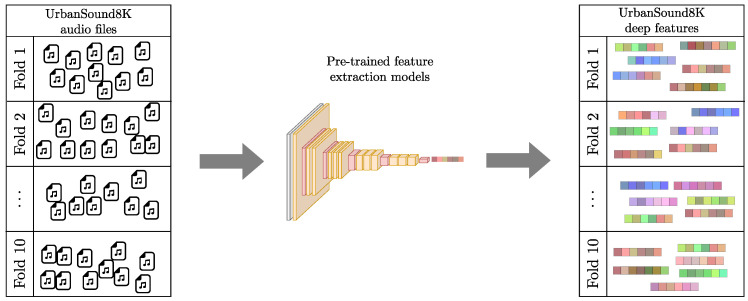
Feature extraction scheme. The audio files from each fold of the UrbanSound8K dataset were characterized using pre-trained models.

As a first step, we employed the VGGish, PANNs, and YAMNet pre-trained models to extract features from the audio files in both datasets to be used as node embedding vectors. In the UrbanSound8K dataset, fold information was preserved for the extracted features, as shown in Figure 3. VGGish model generates a 128-dimensional deep feature vector for every 0.96 s of an audio clip, and YAMNet produces a 1024-dimensional deep feature vector for every 0.48 s. Since the audio files have a maximum duration of four seconds for UrbanSound8K and 60 s for Rey Zamuro, to obtain node embeddings of the same length, we averaged those 128-dimensional VGGish-based and 1024-dimensional YAMNet-based deep features.

Subsequently, for each dataset characterized using the pre-trained models, we constructed a graph where the nodes represented the audio embeddings, and the edges were defined by applying the *k*-NN algorithm, where each node is connected with its *k* nearest neighbors. The value *k* was optimized for each architecture using Optuna [51]. Then, we implemented the GCN, GraphSAGE, and GAT architectures using PyTorch Geometric [52]. For the GCN and GraphSAGE models, we employed a two-layer architecture with a hidden dimension optimized by Optuna and an output dimension equal to the number of classes, i.e., three for the Rey Zamuro dataset and ten for UrbanSound8K. For the GAT model, we used a two-layer architecture, with the first layer having a value for hidden dimension optimized by Optuna and 10 heads, followed by a second layer with an output dimension corresponding to the number of classes and one head.

To compute the attention coefficients, we employed a slope of 0.2 on the LeakyReLU activation function in Equation (5). For all trained GNNs, we used the ReLU activation function and a dropout with a probability of 0.5. All models were trained to minimize cross-entropy loss using the Adam optimizer (with a learning rate of 0.001 and weight decay of $5\times 10^{-4}$) for 300 and 1300 epochs for UrbanSound8K and Rey Zamuro dataset, respectively.

Finally, for UrbanSound8K, we evaluated the performance of the models in terms of accuracy using ten-fold cross-validation, i.e., following the dataset’s distribution across the ten predefined folds. Alternatively, due to the large amount of data and the associated computational cost for training use, the performance of the models for the Rey Zamuro dataset was evaluated with the test set.

## 5. Results and Discussion

Table 1 and Table 2 present the accuracy results of the three GNN models (GCN, GraphSAGE, and GAT) trained for audio file classification, with nodes representing the audio data in a graph. These nodes are characterized by three distinct feature sets derived from pre-trained models (VGGish, PANNs, and YAMNet) applied to UrbanSound8K and Rey Zamuro datasets. Additionally, the tables display the optimal hyperparameters determined by Optuna for each GNN model and node characterization combination. For the UrbanSound8K dataset, where fold distribution is predefined, accuracy results are presented as mean values accompanied by their corresponding standard deviations. Conversely, accuracy results for the Rey Zamuro dataset focus solely on the test set.

The results reveal the consistent superiority of PANNs across both datasets and all three trained GNN models. In particular, on the Rey Zamuro dataset, PANNs show a significant improvement of up to 18% in accuracy. The higher performance can be attributed to the larger dimensional feature space produced by PANNs, with 2048 dimensions, compared to VGGish and YAMNet, which have dimensions of 128 and 1024, respectively. This larger feature space of PANNs is more suitable for capturing detailed information from audio data.

Furthermore, among the compared GNN models, GAT emerges as the top performer, demonstrating sustained superiority across both datasets. This underscores the effectiveness of the attention mechanism in exploiting graph information and optimizing aggregation strategies. Table 3 and Table 4 present the computational costs of the experiments conducted, measured in terms of time and the number of trainable parameters of the networks for the UrbanSound8K and Rey Zamuro datasets, respectively. It is important to note that each model possesses a different number of neurons in the hidden layer due to the optimization performed with Optuna. The GAT model has the highest number of parameters for both datasets and the feature sets generated with the pre-trained models. Specifically, the largest GAT model for the UrbanSound8K dataset has 8M parameters when using PANNs’ deep features. Regarding training time, the GAT model for this dataset can take up to 35 times longer than training GCN and GraphSAGE models. Concerning the Rey Zamuro dataset, we also calculate the time for each model under test. Once again, the GAT model demonstrates the largest number of parameters, as well as longer training and testing times. However, during testing, the times are closer to those of the other two models. Although training time can indeed be long, it is worth considering that a trained network can be scalable regardless of the amount of data. However, it is crucial to consider the computational requirements for building and storing the graph.

Our results show that representing audio datasets through graphs and using deep features extracted from pre-trained models as node features enables sound classification. However, it is important to acknowledge an ongoing research challenge in the graph-building step, particularly in setting its node feature information and edges. To the best of our knowledge, only one study has employed GNNs for sound classification on UrbanSound8K dataset [34]. In one such study, the overall classification accuracy obtained using GNNs was 63.5%, which improved to 73% when GNNs were used in combination with features learned from a CNN. However, our results surpass this, even in the case of GraphSAGE, whose lowest accuracy is 76% for VGGish features. Moreover, our findings are comparable to those reported in other studies employing 1D CNN models. For example, in [18], RawNet CNN was presented, which worked with the raw waveform and achieved an accuracy of 87.7±0.2. Additionally, in [19], a CNN called EnvNet-v2 obtained an accuracy of 78.3%, in [20] with very deep 1D convolutional networks a maximum accuracy of 71.68% only for the 10th fold used as the test set, while in [21], a proposed end-to-end 1D CNN achieved 89% accuracy. In addition, 2D CNN models have also been used on the UrbanSound8K dataset, reaching 79% [22], 70% [23], 83.7% [24], and 97% [25]. It should be noted that although other studies used the UrbanSound8K dataset to train 1D or 2D CNNs, they often employ unofficial random splits of the dataset, conducting their own cross-validations or training-test splits. This causes them to use different training and validation data than published papers that follow the official distribution, making comparison unfair.

## 6. Conclusions

In this paper, we explored using graphs as a suitable representation of acoustic data for sound classification tasks, focusing on the UrbanSound8K dataset and a passive acoustic monitoring study. Particularly, this study offers novel insights into the potential of graph representation learning methods for analyzing audio data.

First, we utilized pre-trained audio models, namely VGGish, PANNs, and YAMNet, to compute node embeddings and extract informative features. Then, we trained GCNs, GraphSAGE, and GATs and evaluated their performance. For the UrbanSound8K dataset, we employed a ten-fold cross-validation approach with the dataset’s predefined folds for performance evaluation. Additionally, we partitioned the Rey Zamuro Dataset into train and test sets to validate its results. Moreover, during the training stage, we conducted hyperparameter optimization to attain the best possible model for the built graphs.

Our findings demonstrate the effectiveness of using graphs to represent audio data. In addition, they show that GNNs can achieve a competitive performance in sound classification tasks. Most notably, it is shown that it is possible to identify ecosystem states through audio and GNNs. Notably, the best results were obtained when employing PANNs-based deep features with the three GNN models. Among the GNN models, the GAT model outperforms the others. This advantage stems from its attention-based operation, enabling it to aggregate node information by assigning weights to its neighbors based on relevance.

To further our research, we plan to explore the feasibility of using temporal GNNs for sound classification tasks to leverage graphs constructed using deep features based on temporal segments of the audio signal, such as those obtained with VGGish and YAMNet. Additionally, the proposed methodology will be applied to the area of soundscape ecology, seeking to generate acoustic heterogeneity maps from the treatment of large volumes of data with GNN techniques that allow exploiting the acoustic relationships between different recording sites.

## Figures and Tables

**Table 1:** UrbanSound8K accuracies.

Feature Model	GNN Architecture	Best Hyperparameters	Accuracy
VGGish	GCN	*k* = 10	0.77 ± 0.04
		n_hidden = 55	
	GraphSAGE	*k* = 12	0.76 ± 0.05
		n_hidden = 57	
	GAT	*k* = 9	0.79 ± 0.05
		n_hidden = 52	
YAMNet	GCN	*k* = 5	0.81 ± 0.04
		n_hidden = 196	
	GraphSAGE	*k* = 11	0.8 ± 0.03
		n_hidden = 55	
	GAT	*k* = 6	0.82 ± 0.04
		n_hidden = 252	
PANNs	GCN	*k* = 4	0.83 ± 0.03
		n_hidden = 40	
	GraphSAGE	*k* = 5	0.82 ± 0.03
		n_hidden = 183	
	GAT	*k* = 10	0.83 ± 0.03
		n_hidden = 206	

**Table 2:** Rey Zamuro accuracies.

Feature Model	GNN Architecture	Best Hyperparameters	Accuracy
VGGish	GCN	*k* = 5	0.63
		n_hidden = 48	
	GraphSAGE	*k* = 10	0.63
		n_hidden = 63	
	GAT	*k* = 6	0.63
		n_hidden = 49	
YAMNet	GCN	*k* = 5	0.76
		n_hidden = 62	
	GraphSAGE	*k* = 6	0.74
		n_hidden = 56	
	GAT	*k* = 6	0.78
		n_hidden = 53	
PANNs	GCN	*k* = 6	0.87
		n_hidden = 64	
	GraphSAGE	*k* = 7	0.85
		n_hidden = 63	
	GAT	*k* = 5	0.91
		n_hidden = 51	

**Table 3:** Computational cost for UrbanSound8K dataset tests.

Feature Model	GNN Architecture	# Parameters	Training Time [s]
VGGish	GCN	7655	13.3
	GraphSAGE	15,799	10.9
	GAT	145,640	86.7
YAMNet	GCN	202,870	18.6
	GraphSAGE	113,805	33.0
	GAT	5,221,480	370.9
PANNs	GCN	82,370	12.7
	GraphSAGE	753,421	37.1
	GAT	8,487,240	453.9

**Table 4:** Computational cost for Rey Zamuro dataset tests.

Feature Model	GNN Architecture	# Parameters	Training Time [s]	Test Time [ms]
VGGish	GCN	6682	14.3	4.9
	GraphSAGE	17,461	25.8	12.9
	GAT	137,240	232.9	84.0
YAMNet	GCN	64,180	20.9	6.1
	GraphSAGE	115,874	68.4	80.4
	GAT	1,098,200	295.4	99.9
PANNs	GCN	131,786	27.2	7.8
	GraphSAGE	259,381	136.9	190.6
	GAT	2,101,240	325.7	120.3

## Data Availability

The data presented in this study are available under request.
